# Diagnosis of a large cystic teratoma of accessory ovary complicated with torsion by ultrasound: A case report

**DOI:** 10.1097/MD.0000000000039716

**Published:** 2024-09-13

**Authors:** Yeping He, Xiaofang Yan, Jianfeng Guo

**Affiliations:** a Department of Ultrasound, Yixing People’s Hospital, Wuxi, Jiangsu, China; b Department of Gynaecology and Obstetrics, Yixing People’s Hospital, Wuxi, Jiangsu, China.

**Keywords:** accessory ovary, ovarian torsion, teratoma, ultrasonography

## Abstract

**Rationale:**

An accessory ovary complicated by cystic teratoma and torsion is extremely rare and requires prompt diagnosis and surgical treatment. However, evidence for effective preoperative imaging diagnosis has barely been reported. Our study presented a case in which preoperative ultrasound reasonably suspected ovarian tumor torsion and an accessory ovary, and laparoscopic surgery was strategically performed.

**Patient concerns:**

An 18-year-old girl had persistent pain in the lower right abdomen for over 7 hours accompanied by nausea and vomiting, and she had a 14.1 × 10.1 × 9.0 cm hypo-echoic cystic lesion containing a 6.4 × 4.9 × 3.0 cm solid component accompanied by the whirlpool sign on the right side of the pelvis. Additionally, a hyper-echoic ovary with a size of 2.5 × 1.4 cm and a normal ovary appearance of 2.4 × 0.8 cm were detected on the right side of the adnexal area by ultrasound.

**Diagnosis:**

The cystic lesion was a large accessory ovarian cystic teratoma, complicated by torsion. The hyperechoic ovary appears as accessory ovarian stromal edema and the normal ovary appearance is eutopic.

**Interventions:**

Single-port laparoscopic resection of the ovarian lesion, release of the ovarian torsion, and oophoroplasty were performed.

**Outcomes:**

Postoperative recovery was unremarkable. Antral follicles were detected in both eutopic and accessory ovaries by ultrasound 20 days and 4 months after surgery. In addition, during the second postoperative ultrasound follow-up, the accessory ovary showed no difference in echo compared to the normal ovary, except for a slightly larger volume.

**Lessons:**

Clinical manifestations of accessory ovarian tumors combined with torsion are similar to those of eutopic ovarian torsion, and timely surgery is required.

## 1. Introduction

The accessory ovary is a rare developmental abnormality in the female reproductive system, and that is particularly rare complicated by large cystic teratomas and torsion, which may be life-threatening.^[1]^ So, it is difficult to monitor the disease and perform surgery in a timely manner. Therefore, the treatment of various comorbidities of accessory ovaries, such as tumors, remains a clinical challenge. Herein, we report a case of a large cystic teratoma arising from an accessory ovary complicated with torsion that was diagnosed preoperatively using ultrasonography and present a comprehensive discussion and summary of this case combined with a literature review.

The institutional review board of Yixing People’s Hospital approved this study, and written informed consent was obtained from the patient for the publication of this case report.

## 2. Case presentation

**Figure 1.**
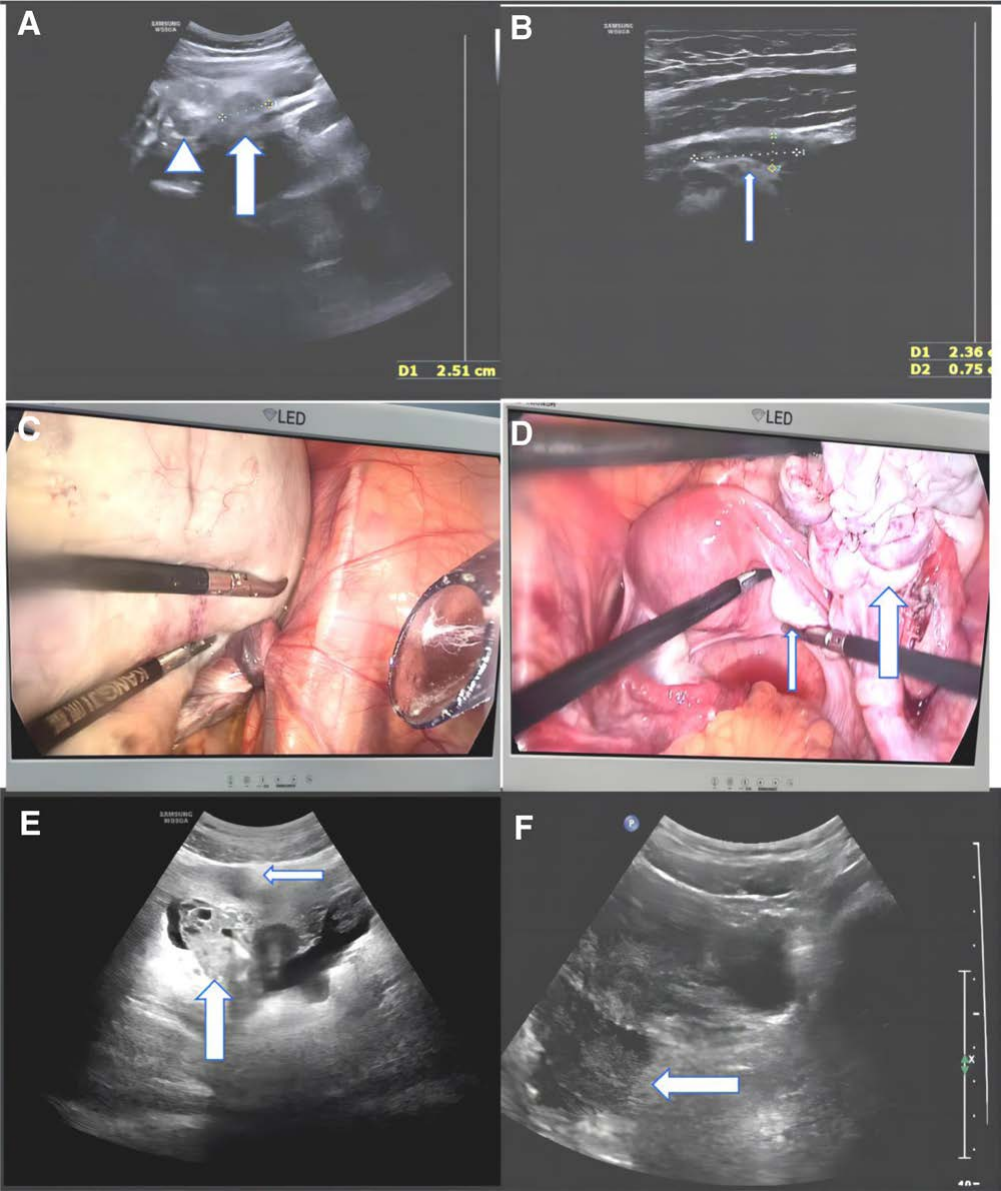
Preoperative ultrasound image showed a hyper-echoic ovary appearance of 2.5 × 1.4 cm size (A) and a normal ovary appearance of 2.4 × 0.8 cm size (B) on the right adnexal area. Intraoperative findings showed the accessory ovarian tumor with torsion (C) and the eutopic right ovary (D). Postoperative ultrasound image showed hypo-echoic eutopic ovary and hyper-echoic accessory ovary 20 d after the surgery, antral follicles were found both in them (E). Ultrasound result 4 mo after the surgery: Two hypo-echoic findings on the right side adnexal area: echoic eutopic ovary and accessory ovary (F). The thin arrow, the eutopic right ovary. The thick arrow, the accessory ovary. The arrowhead, the whirlpool sign.

An 18-year-old girl, unmarried and nulliparous, was admitted to a local hospital because of persistent pain in the lower right abdomen for over 7 hours, accompanied by nausea and vomiting once in September 2023. Ultrasonography and computed tomography (CT) both revealed a pelvic mass. Subsequently, the patient was referred to the gynecology ward of our hospital. Physical examination showed abdominal distension and tenderness in the lower abdomen, no rebound pain, vital signs, pregnancy test, blood tests and tumor marker tests were within normal parameters. Due to the patient’s lack of sexual history, the gynecologist conducted a transanal examination and palpable a 15 × 15 cm mass on the right side of the pelvic cavity, which was fixed with high tension and tenderness. There has been no such history. Transabdominal sonography showed a 14.1 × 10.1 × 9.0 cm hypo-echoic cystic lesion containing 6.4 × 4.9 × 3.0 cm solid component in the right side of the pelvic, anterior to the uterus, the right anterior wall of the cyst is locally thickened, the whirlpool sign and ovarian stromal edema were seen with a size of 2.5 × 1.4 cm (Fig. 1A). However, a normal ovary appearance (2.4 × 0.8 cm) was detected behind and above the tumor (Fig. 1B). Additionally, little free fluid was detected in the pouches of Douglas. The left ovary and uterus were unremarkable.

Although there was little confusion regarding the appearance of the 2 ovaries (we could not confirm which was the normal ovary), an emergency laparoscopic examination was performed because of ovarian cyst torsion. Approximately 24 hours from the onset of abdominal pain to the start of surgery. Single-port laparoscopy revealed an ovarian cyst with a smooth surface and good color, measuring approximately 14 × 13 cm in diameter and twisted 720° at the pedicle (Fig. 1C). The surface cortex of the ovarian cyst was cut open, the cortex and cyst walls were separated, the ovarian cyst wall was completely peeled off, and the cyst ruptured unintentionally during manipulation, showing sebum and hair components. After cystectomy, it was found that the cyst was adjacent to the fimbriae of the fallopian tube, arising from the accessory ovary, and a normal-appearing right ovary was observed (Fig. 1D). The fallopian tubes, uterus, left ovary, kidneys, and ureters were all normal. Oophoroplasty was performed on the accessory ovary (Fig. 1D). Postoperative pathological examination revealed cystic teratoma. The entire surgery lasted approximately 170 minutes. The patient recovered well, and was discharged on the 5th day after surgery.

The first follow-up was performed 20 days after surgery. Ultrasound imaging showed that the echo of the accessory ovary was stronger than that of the normal ovary, and antral follicles were observed in both the right orthotopic and accessory ovaries (Fig. 1E). A second follow-up was conducted at 3-month intervals, and the volume of the accessory ovary was still slightly larger; however, the echo did not differ from that of the normal ovary (Fig. 1F).

## 3. Discussion

### 3.1. Accessory ovary

The accessory ovary is situated near the normal ovary and connected with it according to the location of the ovaries and contains ovarian tissue,^[1]^ however considering that no classification can be completely rigid, we searched the PubMed and China National Knowledge Infrastructure databases from construction to March 1, 2024, using “torsion” and “accessory ovary” or “supernumerary ovary” or “ectopic ovary” as keywords, only a few cases which were complicated with torsion.^[2–4]^ As far as our knowledge, this is the first reported case of accessory ovarian cyst torsion diagnosed using ultrasonography before surgery.

The incidence of accessory ovaries in the normal population is unknown, and cases of accessory ovaries that are ever recognized are those discovered incidentally during operations for unrelated conditions or those that developed pathological, symptomatic disorders such as tumors or autopsy, with an incidence of approximately 1/93 000 in gynecologic hospital admissions, as reported by Wharton.^[1]^ The accessory ovaries are usually smaller than 1 cm in size, but there are also some accessory ovaries that are the same size as the normal ovary and have no abnormalities on biopsy.^[1,5]^ The accessory ovary may develop from abnormal embryogenesis or ovarian implants^[1,6,7]^ such as postsurgical or postinflammatory implants, and nearly 50% of patients with accessory ovaries have a history of pelvic surgery. 26% to 36% of cases of accessory ovary have associated congenital defects that are probably much higher than those in normal persons, such as accessory fallopian tube, accessory adrenal gland, and lobulated liver.^[1,8]^ In our case, no associated anomaly was found, and we believe it to be a true embryonic accessory ovary because there was no history of pelvic disease or surgery.

### 3.2. Accessory ovary combined with tumor

The accessory ovary may have the same function as the normal ovary because it contains ovarian follicular tissue.^[1]^ Thus, any tumor occurring in the normal ovary can also arise from the accessory ovary, although they are even rarer. There are various types of accessory ovarian tumors such as endometriosis cyst, dermoid cyst, serous cystadenomas, steroid cell tumor, Brenner tumor, and sclerosing stromal tumor.^[3–5,9–12]^ The most common type is teratoma, which accounts for approximately 60% of all cases.^[11]^ It is often asymptomatic, with abdominal distention and occasional pelvic pain being the most common symptoms. Similar to eutopic ovarian tumors, accessory ovarian tumors are usually treated surgically. Most patients have good outcomes when choosing the appropriate surgical timing. Recurrent bilateral dermoid cysts have been reported 12 years after the first surgical resection of accessory ovaries.^[13]^

### 3.3. The torsion of accessory ovarian tumor

The torsion of accessory ovarian tumors is extremely rare. In our review of the PubMed literature, we found 3 cases of accessory ovarian torsion, all of which were accompanied by tumors, 1 case of endometriotic cyst, 1 case of dermoid cyst, and the third case of a benign tumor without a specific pathological type. In addition to our case, all 4 patients had abdominal pain accompanied by nausea and vomiting, similar to eutopic ovarian torsion. Common ultrasound signs include adnexal enlargement, ovarian interstitial edema, the whirlpool sign, and pelvic fluid accumulation.^[14]^ Ovarian torsion is an emergency that requires timely diagnosis and treatment, because ovarian loss can have long-term consequences in terms of fertility. In this case, timely surgical removal of the accessory ovarian lesion, release of torsion, and oophoroplasty was relatively safe.

### 3.4. Diagnosis of the accessory ovary

The preoperative diagnosis of the accessory ovary is notoriously challenging. Kosasa et al preoperatively diagnosed the supernumerary ovary with human chorionic gonadotropin in a woman who had undergone bilateral oophorectomy and adrenalectomy for breast cancer.^[15]^ This case was diagnosed before operation by ultrasonography, and after surgery, the ultrasound examination showed active ovarian tissue both in the right normal and accessory ovaries. This may support the notion that the accessory ovary has a functional capability and can be preserved in women with fertility requirements. However, this may also be a limitation of this case report, as the discovery of antral follicles does not directly prove the reproductive function of the accessory ovary. This finding warrants further investigation. Another limitation of this study is the lack of other imaging diagnostic data. The patient did not undergo magnetic resonance scan, and CT scan was completed in the local hospital, making it impossible for us to show the CT imaging findings.

## 4. Conclusion

Although extremely rare, an accessory ovarian tumor complicated by torsion should be considered if there is evidence, and emergency surgery should be performed as in a eutopic ovary.

## Author contributions

**Investigation:** Yeping He, Xiaofang Yan.

**Methodology:** Yeping He, Jianfeng Guo.

**Project administration:** Jianfeng Guo.

**Resources:** Jianfeng Guo.

**Supervision:** Jianfeng Guo.

**Visualization:** Yeping He, Xiaofang Yan.

**Writing – original draft:** Yeping He, Xiaofang Yan.

**Writing – review & editing:** Yeping He, Xiaofang Yan, Jianfeng Guo.
